# Evaluation of medical decision support systems (DDX generators) using real medical cases of varying complexity and origin

**DOI:** 10.1186/s12911-022-01988-2

**Published:** 2022-09-24

**Authors:** P. Fritz, A. Kleinhans, R. Raoufi, A. Sediqi, N. Schmid, S. Schricker, M. Schanz, C. Fritz-Kuisle, P. Dalquen, H. Firooz, G. Stauch, M. D. Alscher

**Affiliations:** 1 Department of Pathology, Robert-Bosch-Hospital, 70376 Stuttgart, Germany; 2 IPath Telemedicine Network Gemeinnützige GmbH, 26603 Aurich, Germany; 3 Abu Ali Sina Hospital, 1702 Mazari al Sharif, Afghanistan; 4 Robert Bosch Gesellschaft Für Medizinische Forschung mbH, Stuttgart, Germany; 5 Department of Internal Medicine and Nephrology, Robert-Bosch-Hospital, Auerbachstr. 110, 70376 Stuttgart, Germany; 6 Department of Anesthesia, Kreiskrankenhaus Günzburg, 89312 Günzburg, Germany; 7 Institute of Pathology University Basel, 4031 Basel, Switzerland; 8 Firooz Medical Laboratory, 3001 Herat, Afghanistan; 9 Robert-Bosch-Hospital, Management board, 70376 Stuttgart, Germany

**Keywords:** Medical decision support systems (MDSS), Telemedicine, Second opinion, Diagnosis assistance systems, CDSS, DDx generator

## Abstract

**Background:**

Medical decision support systems (CDSSs) are increasingly used in medicine, but their utility in daily medical practice is difficult to evaluate. One variant of CDSS is a generator of differential diagnoses (DDx generator). We performed a feasibility study on three different, publicly available data sets of medical cases in order to identify the frequency in which two different DDx generators provide helpful information (either by providing a list of differential diagnosis or recognizing the expert diagnosis if available) for a given case report.

**Methods:**

Used data sets were *n* = 105 cases from a web-based forum of telemedicine with real life cases from Afghanistan (Afghan data set; AD), *n* = 124 cases discussed in a web-based medical forum (Coliquio data set; CD). Both websites are restricted for medical professionals only. The third data set consisted 50 special case reports published in the New England Journal of Medicine (NEJM). After keyword extraction, data were entered into two different DDx generators (IsabelHealth (IH), Memem7 (M7)) to examine differences in target diagnosis recognition and physician-rated usefulness between DDx generators.

**Results:**

Both DDx generators detected the target diagnosis equally successfully (all cases: M7, 83/170 (49%); IH 90/170 (53%), NEJM: M7, 28/50 (56%); IH, 34/50 (68%); differences n.s.). Differences occurred in AD, where detection of an expert diagnosis was less successful with IH than with M7 (29.7% vs. 54.1%, *p* = 0.003). In contrast, in CD IH performed significantly better than M7 (73.9% vs. 32.6%, *p* = 0.021). Congruent identification of target diagnosis occurred in only 46/170 (27.1%) of cases. However, a qualitative analysis of the DDx results revealed useful complements from using the two systems in parallel.

**Conclusion:**

Both DDx systems IsabelHealth and Memem7 provided substantial help in finding a helpful list of differential diagnoses or identifying the target diagnosis either in standard cases or complicated and rare cases. Our pilot study highlights the need for different levels of complexity and types of real-world medical test cases, as there are significant differences between DDx generators away from traditional case reports. Combining different results from DDx generators seems to be a possible approach for future review and use of the systems.

**Supplementary Information:**

The online version contains supplementary material available at 10.1186/s12911-022-01988-2.

## Background

Clinical decision systems (CDSS) are increasingly used in practice [1–4]. However, all new methods in medicine, whether diagnostic or therapeutic, must be tested to demonstrate feasibility of use and benefit to patients. Most currently approved CDSS are limited to clinically well-defined situations, such as detecting early signs of deterioration in a postanesthesia care unit [5] or distinguishing melanocytic lesions in melanoma from nevi [6]. However, one hope in using CDSS systems is to use them more broadly, for example, to reduce misdiagnosis, shorten diagnosis times, and prevent rare diseases from being forgotten in the differential diagnosis list [1, 7–11]. In this context, CDSS systems that are not designed to address a single question but to output possible differential diagnoses (all diseases and symptoms are allowed) are referred to as differential diagnosis generators (DDx) [12]. Nevertheless, testing these approaches is complicated by several problems: (1) The gold standard (ground truth or true diagnosis) is often unknown [2, 7, 8]. At best, a group of experts has made a diagnosis that can be used as the gold standard or target diagnosis. Further, it is difficult to determine the usefulness of even inapplicable but important differential diagnoses (e.g., exclusion of possible other diseases or combinations). Moreover there is no clear protocol on how to test such systems in terms of case complexity or testing scenario in a holistic context.

To the authors' knowledge, there are only a few publications that have examined the performance of individual DDX generators (summarized in [13]). Even fewer studies that have compared the performance of multiple DDX generators and their results using test data [12]. In addition, the lack of complex cases in the aforementioned meta-analysis has been criticized [13] and comparability due to heterogeneous testing approaches is not given.

Therefore, comparative studies with concrete examples from clinical routine in different complexity levels are still missing. And this despite the fact that these systems have been described for decades [14]. Like any other medical technology or intervention, diagnostic tools should be evaluated before being introduced into daily practice [15, 16]. Fittingly, the U.S. National Academy of Medicine has recently highlighted that evidence on the performance of DDX tools in routine clinical practice is currently lacking and called for more research on CDSS, and specifically DDX, tools in real-world settings and the comparison and validation of different implementation models [16, 17].

To test the utility of DDX's, one should look at the requirements of potential users of such systems. Therefore, we identified three different situations with the need of support in differential diagnosis considerations: (1) situations of limited resources, where sophisticated investigation methods are not available and medical education may be limited. (2) primary care provider who are confronted with patient symptoms at a first contact that are difficult to interpret. (3) the most complex cases and rare or orphan disease.

Derived from this, we pursued a threefold goal: First, we wanted to compare two diagnostic systems (IsabelHealth and Memem7) on the basis of real test cases in order to highlight possible differences. Second, we wanted to expose the systems to unstructured cases of varying complexity to describe their usefulness with respect to the scenarios described above. Third, we would like to initiate a scientific discussion on possible methods to compare and validate DDXs in the future. Here we report on some metrics that allow an initial evaluation of such systems and publish a test data set that also allows the scientific public to compare them with other systems.

## Methods

### Principle structure of the feasibility study

We used three test datasets (105 cases from a telepathology platform from Afghanistan (Data Set I), 124 cases on a medical discussion platform (Coliquio, Data Set II), and 50 case reports taken from New England Journal of Medicine (NEJM, Data Set III) (following section and supplement for details and references (Additional file 1: Table S1)). DDx used were IsabelHealth and Memem7.

**Fig. 1:**
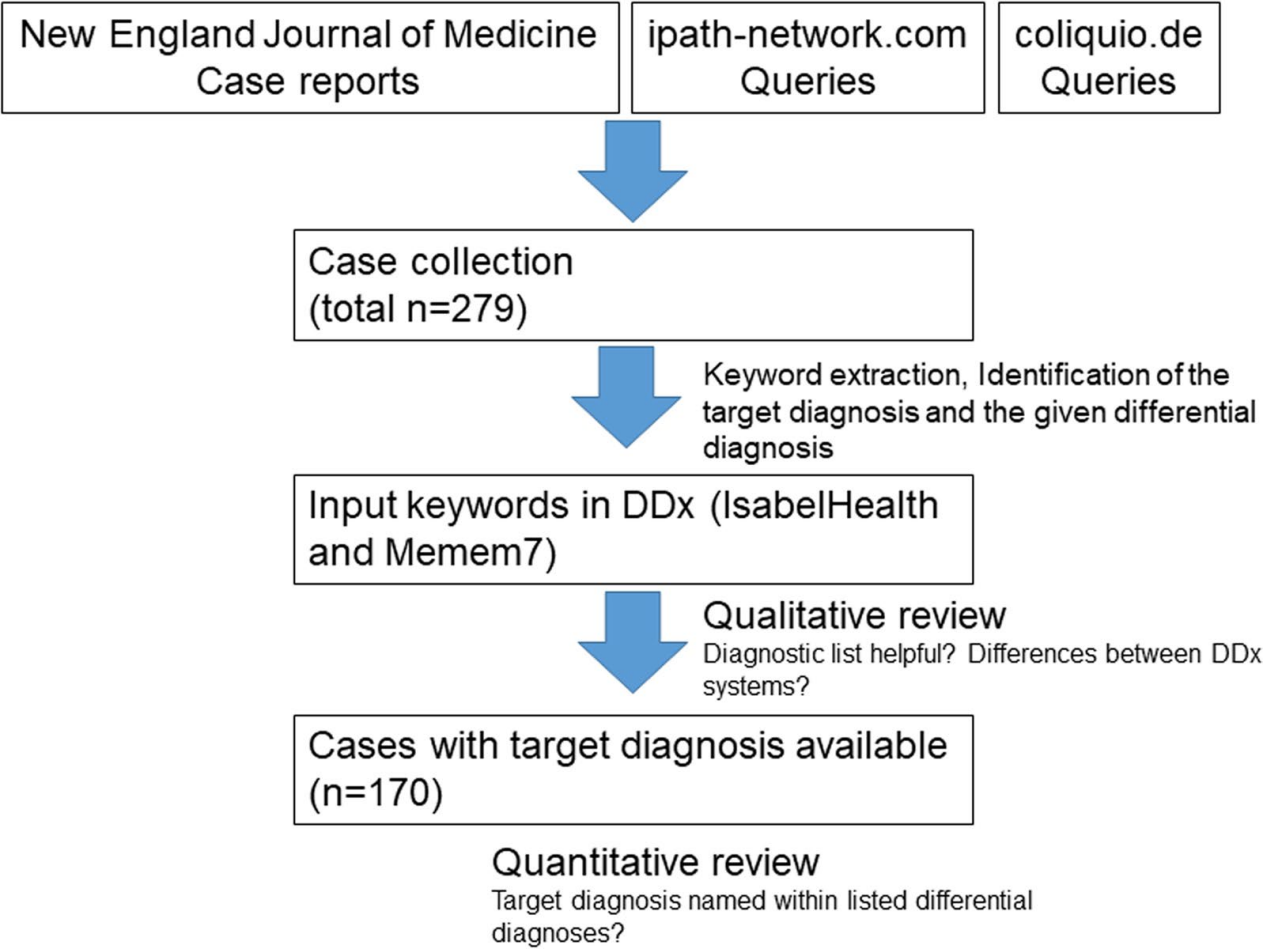
Study protocol. DDx = Differential diagnosis generators

The same test strategy was adopted for all three dataset (see also Fig. 1).

The terms used for the search function in Memem7 and IsabelHealth were chosen by one author (SS). Target diagnosis for the Afghan dataset was the opinion of at least three experts (senior pathologists). For the Coliquio dataset, the target diagnosis was the differential diagnosis favored by the majority of discussants, and for the New England Journal of Medicine, the gold standard was the principal diagnosis (including 5 differential diagnoses) proposed by the authors of the case report. In the Afghan and Coliquio cases, a principal diagnosis was not available from an expert in all cases. Two authors (PF and CF) evaluated the issued differential diagnoses with regard to their helpfulness (“helpful”/“not helpful”).

### Datasets

Dataset I (Afghanistan): This dataset consists of 105 ongoing medical cases acquired from a tele-pathology platform (ipath [18, 19]) in 2017–2019 with daily diagnostic use for patients treated in Afghanistan (Mazar al Sharif). Each test case was diagnosed based on clinical and morphological data. Responsible for these test cases were three primary physicians in Mazar al Sharif, Afghanistan (RR, AS, HF), the diagnosis was made by four international senior experts (PF, GS, PD, BS). Unlike the other datasets, the cases in this series were dominated by morphological descriptors and questions. The Afghan test set represents user requests from physicians in a country with limited resources. In most cases, there is a lack of sophisticated testing methods such as specialized laboratory methods, immunohistochemistry, or imaging.

Dataset II (Coliquio test cases) [20]: The 124 cases were collected between 2018 and 2019. Coliquio is a German-language online expert network that specializes in knowledge exchange for physicians. Only licensed physicians and licensed psychotherapists have access. Aim of the platform is to exchange information on patient cases, diagnoses and therapy options. Cases were screened in chronological order after creation. A case was included as a test case if (1) at least two symptoms were reported by the physician presenting the case in the Coliquio forum and if (2) both sex and age information were provided. The Coliquio dataset was dominated by clinically oriented descriptions of a patient. The query from this user group reflects the situation of a primary care physician who is treating a difficult case. She/he may be looking for an alternative explanation for the patient's symptoms.

Data set III (NEJM): 50 cases from the New England Journal of medicine were chosen. For each case the article provided an expert diagnosis and five differential diagnosis. The references can be found in the additional file (Additional file 1: Table S1).

The used dataset of keywords and target diagnoses of all test cases can also be found in the additional file (Additional file 1: Table S2).

*Examples of test cases* For a better understanding of the study approach three test cases, one of each data set, were randomly selected and are described in Tables 3, 4, 5.

### Used software systems

Isabel Health [21]: IsabelHealth is a commercial DDX generator built using machine learning technology [4]. It is a "black box" system for the user, where the thesauri used cannot be reviewed or improved by the tester. For each case, a search function was available with up to 10 symptoms, and for the allowed terms IsabelHealth provides a thesaurus. From these, a ranked list of 100 possible differential diagnoses is generated in descending probability. Only the first 10 diagnoses were considered for the evaluation.

Memem7 [22–24]: Is a currently non-commercial DDX developed by two of the authors (KA, PF). Memem7 is based on a large semantic network (about 560,000 nodes) that is transparently represented to the user, containing all kinds of entities and relationships such as objects, classes, parts, attributes, processes, states, properties, etc. The inference algorithms use the processing of the semantic network based on linguistic logic, which includes ambiguity, vagueness and uncertainty. For each case, a search function can be used based on the terms entered. The input is mainly structured, but unstructured narrative input (e.g., medical reports) is also possible, which is processed by modified NLP algorithms. The results are output as a ranked list of possible differential diagnoses with no length restriction. For each diagnosis, Memem7 provides a relevance value indicating the relevance of the search terms to the proposed diagnosis. Bayesian methods are used for diagnosis ranking: The more the terms match leading symptoms, the higher the relevance value.

### Statistical methods

Excel was used for data collection and the statistical package R (version 3.5.3) [25] for statistical analysis. Statistical significance was assumed for *p* < 0.05. Numerical data were analyzed with the t-test and factors with the chi-square test.

### Ethical aspects

All data used are anonymized, i.e. they cannot be attributed to patients in any way. For the Coliquio cases, neither date of birth, name nor place of residence were given. For the Afghan cases, all cases in the ipath network are anonymized by the responsibility of the treating physician. Only the hospital where patients were treated, but neither name nor date of birth is known. All NEJM test cases are published and therefore ethical aspects are the responsibility of the publishing authors.

## Results

The Afghan test cases consist of every day cases and rarely any complicated cases, but contain a high number of describing morphological terms. The Coliquio test cases are more complicated cases, often missing an expert diagnosis. The NEJM test cases were mostly highly complicated cases.

The cases covered nearly the whole spectrum of medicine (Table 1) with only few cases of a psychiatric background.

**Table 1:** Distribution of the test cases in relation to the medical specialty

Medical discipline	Number	%
Internal Medicine	44	15.7
Hematology/Oncology	37	13.2
Gynecology	34	12.2
Dermatology	30	10.8
Gastroenterology	24	8.6
Orthopedics/Rheumatology	22	2.2
Neurology	18	7.9
Infectiology	17	6.5
Dentistry	14	6.1
Cardiology	10	5.0
ENT (Ear, Nose and Throat Medicine)	9	3.2
Nephrology/ Urology	7	2.5
Pulmonary medicine	6	2.2
Endocrinology	4	1.4
Psychiatry	2	0.7
Ophthalmology	1	0.4

Patients in Afghan test sets are significantly younger (*p* = 0.005 and *p* = 0.01) as compred with NEJM or Colliquio patients (Table 2).

**Table 2:** Description of test cases with concern to age, used terms and sex

Variable	Mean	SD	Median
Age (*n* = 279, all cases)	40.2	23.4	38
Afghan (*n* = 105)	35.1	22.4	30
Colliquio (*n* = 124)	42.7	22.2	40
NEJM (*n* = 50)	44.2	16.5	42.5
Number of terms (all cases)	7.0	3.69	7.0
Afghan	7.0	3.2	7.0
Coliquio	6.8	3.9	7.0
NEJM	7.4	4.1	7.5

	Female	Male
Sex distribution (*n* = 279, all cases)	*n* = 136, 57%	*n* = 103, 43%
Afghan (*n* = 105)	*n* = 52, 68%	*n* = 25, 32%
Colliquio (*n* = 124)	*n* = 60, 54%	*n* = 52, 46%
NEJM (*n* = 50)	*n* = 24, 46%	*n* = 26, 54%

Percentages without missing values, rounded to whole numbers

There were significantly less males in the Afghan and Colliquio test set than in the NEJM data set (*p* = 0.01).

There was no difference between the three data sets for the number of terms extracted to describe the test cases (see Table 2). Note that in the Afghan data sets an expert diagnosis was only available in 74/105 (70.5%) of cases and in the Coliquio data sets in only 46/124 (37.1%) of cases. This low frequency was because an expert diagnosis in the Coliquio data sets was accepted only per protocol if proposed by most discussants. In the NEJM an expert diagnosis was given in all 50 cases by the design of the reports. Only cases with target diagnosis were accepted.

### Qualitative differences in DDx results

In some cases both DDx provided different, yet interesting alternatives to the final expert diagnosis. Table 3 gives an example.

**Table 3:** Example of a test case of the Afghan data set

Case ID	Afghan dataset, Ipath-network_ID 1,128,468, Kasuosom 4362
Symptoms/Terms	Male Aged 21 years Liver cyst Cyst wall fibrosed
Target diagnosis (expert diagnosis)	Liver cyst NOS (unknown cause)
Expert differential diagnoses	Choledochal cyst, echinococcal cyst
Result Memem7 preferred diagnosis	Peribiliary liver cyst
Result Memem7, differential diagnosis	Echinococcal cyst, endometrial liver cyst
Result IsabelHealth preferred diagnosis	Choledochal cyst
Result Isabel, differential diagnosis	Intracranial Hematoma, Endocarditis, Arterial Aneurysms, Brain, Neoplasms, Multiple Sclerosis, Langerhans Cell Histiocytosis Class 1, HIV/AIDS, Adrenal Neoplasms, Leptospirosis, Fibromyalgia

Of note, both DDx confirm the expert diagnosis, but one DDx adds an interesting and valuable DD (endometrial cyst).

Further, our results showed that DDx can add helpful DD for a case and further useful suggestions. Example 2 (see Table 4) shows that the discussants in Coliquio were not able to agree on a preference diagnosis, while both DDX provided a reliable DD for the given test case with probabilities and made further useful suggestions beyond the discussion.

**Table 4:** Example of a test case of the Coliquio dataset

Case ID	Coliquio Case 12
Symptoms/Terms	metal taste in oral cavity, Backpain, tramadol medication, pramipexol, gingivitis, oral metall prothesis, gingivitis
Target diagnosis (expert diagnosis)	n/a (11 discussants without clear majority of opinion)
Expert differential diagnoses	Hashimoto thyroidtis, ADR Ramipril, UAW Tramadol
Result Memem7 preferred diagnosis	no
Result Memem7, differential diagnosis	Pramipexole side effect, Pine nut chewing, Lead poisoning, Mercury intoxication, Intoxication by acetone, Hyperkalemia, Ramipril side effect
Result IsabelHealth preferred diagnosis	Mercury intoxication
Result Isabel, differential diagnosis	Pyorrhea, Gingivitis/Stomatitis, Mercury Intoxication, Sjogren's Syndrome, Heavy Metal Intoxication, HIV / AIDS, Lichen Planus, Interstitial Nephritis, Dental Abscess, Enterovirus Infections

Both examples demonstrate that both systems may point to forgotten DD and combining two DDX systems may broaden the list of DD.

Example 3: (Table 5):

**Table 5:** Example of a test case of the New England Journal dataset

Case ID	NEJM Case 34
Symptoms/Terms	Headache, cognitive changes, chest pain, dysaesthesia nausea, veteran, weigth loss, traumatic injury
Target diagnosis (expert diagnosis)	Post-traumatic stress syndrome
Expert differential diagnoses	Meningitis, encephalitis, pseudotumour cerebri, traumatic brain injury
Result Memem7 preferred diagnosis	no
Result Memem7, differential diagnosis	Encephalitis
Result IsabelHealth preferred diagnosis	Lung Neoplasm
Result Isabel, differential diagnosis	Aortic Aneurysm/Dissection, Hyperthyroidism, Relapsing Polychondritis, Coronavirus, Intracranial Hematoma, Subdural Hematoma, Arterial Aneurysms, Monoclonal Immunoglobulin Deposition Disease,Cirrhosis, Infectious Mononucleosis

This is an example, where both DDx do not recognize the expert diagnosis

The target diagnosis in this case was posttraumatic stress syndrome and both DDx failed to find this expert diagnosis, nevertheless the results were useful and pointed to some interesting DD. This underlines once again that even the DDx systems cannot present a conclusive truth, but are nevertheless helpful in finding a diagnosis and are suitable for excluding e.g. somatic disorders.

In a subjective dichotomous assessment of the usefulness of the differential provided by two authors, both systems performed equally well with the exception of the Afghan dataset, where Memem7 performed significantly better than IsabelHealth (*p* < 0.00001, see Table 6).

**Table 6:** Subjective rating

Data set	DDx	Number of helpful differential diagnoses	%
Afghan *n* = 105	Experts	Not evaluated	
Afghan *n* = 105	Memem7	75	71.4
Afghan *n* = 105	Isabel	28	26.7
Coliquio *n* = 124	Discussants	94	75.8
Coliquio *n* = 124	Memem7	79	63.7
Coliquio *n* = 124	Isabel	81	65.3
NEJM *n* = 50	Two authors	29	58
NEJM *n* = 50	Memem7	32	64
NEJM *n* = 50	Isabel	35	70

Subjective rating by two of the authors of the provided list of differential diagnoses as either "helpful" or "not helpful" either established by the discussion in the respective platform or suggested by the deployed DDx systems

### Quantitative differences of DDx

IsabelHealth provides a list of DD (up to 100) ranked and annotated with red markers for dangerous diseases. Memem7 provides a list of DD in ranked order. The number of proposed DD varies from case to case. Memem7 lacks a system of red flags.

Only cases with target diagnosis were included in the further evaluation. Memem7 performed equally in all three test systems (*p* = 0.43, no significant difference), with respect to the deployment of DD. Interestingly, within the Afghan data set recognition of an expert diagnosis was less successful in IsabelHealth as compared to Memem7 (*p* = 0.003), where Memem7 detected the expert diagnosis in 54.1% versus 29.7% detected by IsabelHealth (Table 7).

**Table 7:** Performance of DDx generators in the recognition of a target diagnosis

Data sets	Abbreviation for statistic	DDX	*N*	%	*p*
Afghan *n* = 74	a	Memem7	40	54.1	*p*_ab_ = 0.07
Afghan *n* = 74	b	IsabelHealth	22	29.7	*p*_ab_ = 0.009
Coliquio *n* = 46	c	Memem7	15	32.6	*p*_cd_ = 0.021
Coliquio *n* = 46	d	IsabelHealth	34	73.9	*p*_cd_ = 0.025
NEJM *n* = 50	e	Two authors	14	28	*p*_ef_ = 0.10, *p*_eg_ = 0.027
NEJM *n* = 50	f	Memem7	28	56	*p*_fg_ = 0.66
NEJM *n* = 50	g	IsabelHealth	34	68	*p*_efg_ = 0.05

In the Coliquio test cases, the IsabelHealth systems performed significantly better than Memem7 with 73.9% and 32.6% respectively (*p* = 0.021) in recognizing the expert diagnosis. Both DDx generators performed equally successful in 56–68% of the NEJM cases. This difference here was not significant. Taken all cases together Memem7 recognized the expert diagnosis in 83/170 (49%) cases versus 90/170 (53%) cases in IsabelHealth. As DDx are black box systems, one future-oriented strategy of testing may be to accept only results, where both DDX yield the same result. This occurs in 46/170 cases corresponding to only 27.1%. of cases with an identified target diagnosis.

### Performance of non expert physicians in comparison with DDx

In order to test the usefulness of the DDx systems in helping physicians determine possible differential diagnosis, two authors (medical doctors, but non-experts, CF, PF) tried to identify the target diagnosis and differential diagnosis of the NEJM test cases. Both performed less successfully than both DDx in recognizing only (14/50) 28% of target diagnosis. The difference to the performance of IsabelHealth was significant (*p* = 0.027).

## Discussion

Here we report on a comparison of two clinical decision support systems (IsabelHealth and Memem7), exposing the systems to three datasets of unstructured cases of varying complexity. Taken together, both systems provided substantial help in finding a list of differential diagnoses (DD) or identifying the target diagnosis in all three test situations, with a slight superiority of IsabelHealth on more complex clinical cases. Across all cases, both DDx generators were subjectively found to be helpful in providing a list of DDs.

Our results, recognizing the expert diagnosis in approximately 50% of all test cases, was somewhat lower (but nevertheless very promising) than those reported in literature [12, 26–32]. Rammarayan et al. [26] claimed to recognize the discharge diagnosis in 95%. With the DDx generator IsabelHealth a 79.5% recognition of rare diseases (orphan diseases) was found by Reumann and coworkers [27]. The reason for the lower results in our study is in our opinion the restriction on the first 10 proposed (and most likely) diagnoses as well as the restriction to routine findings of an initial medical contact. A recent evaluation of DDX in general practice provided evidence that too large a list of (inappropriate) differential diagnoses, may hinder its usefulness in everyday practice [28]. We would also argue that comparisons of test results between studies are limited because the results are highly dependent on the level of detail selected for the clinical information and terms. The published results, mainly with the DDx generator IsabelHealth shows a wide spectrum of evaluations from enthusiastic ones [26] moderately positive ([12, 29], and own experiences) to more sceptic ones [13, 28]. All publications, however, share two points: a) a request for more investigations prior to clinical use and b) recognition of the inherent potential of DDx generators. Comparing both DDx generators and the three user groups Memem7 performed slightly better in the situation of patients treated in a country with restricted resources. IsabelHealth performed better than Memem7 in the situation of patients with very complicated and rare diseases. The contrasting difference in the results of the Afghan test data set, in which Memem7 performed significantly better, cannot be conclusively attributed to the fact that IsabelHealth is a black box system. We explain this difference by the large number of histopathologic, morphologic terms and the platform’s objective (pathology) used in the Afghan dataset. However, we speculate that the history of Memem7 with a special focus on histopathology as a clinical subject resulted probably in a more adapted thesaurus to morphological terms used in Memem7 as in Isabel Health. Examples would be terms regarding, for example, tumor cells: "unicellular"; location: femur, nucleoli: detectable, or similar. This might explain the better performance of Memem7 here.

It should also be noted that even with comparable high detection rates of both systems, the concordance of both systems is quite low with only about 27% matching target diagnosis. This, together with our observation that both systems helpfully complement each other in terms of completeness of a useful lists of differential diagnoses, may suggest that combined use of multiple DDx systems may offer advantages where appropriate.

This study has some limitations that should be considered in the interpretation. First, the influence of variables such as gender and age on your results of the two DDx is not clearly known, since at least Isabel Health is a black box system. Here, uncertainties arose due to the clear differences in gender distribution and the not clearly known age (due to anonymization) in the data sets of ipath (Afghanistan) and Coliquio platforms. Further, although almost all medical specialties were covered by our test sets, the distribution might have played a role regarding the focus of DDx systems and transferability of the performance in other datasets.

In addition, one should keep in mind in the interpretation that CDSS and DDx generators are learning systems (which can be trained, e.g., by unrecognized cases) and studies therefore always represent only a snapshot at the time of registration and might already be outdated again at the time of publication of this study.

Using the TELOS criteria [33] for a feasibility study, we found that both DDx are functional in the sense of providing a helpful listing of differential diagnoses.

We see our approach to validation of CDSS on real clinical questions as an important initiative for a scientific discussion on possible methods to compare and validate DDXs in the future. Like any other diagnostic tool DDXs should be evaluated before being introduced into daily practice [15]. Nevertheless, evidence on the performance of DDX tools in routine clinical practice is still scarce. Not without reason, the U.S. National Academy of Medicine has called for more research on DDx tools in real-world settings and the comparison and validation of different implementation models [16, 17].

Moreover, we provide a defined dataset of real-world cases based on physician queries for future evaluation of DDX systems.

However, the future of using DDx in the clinical setting [13] depends on several unresolved issues: (1) vendors of DDx systems should clearly define the field of application and the limitations of their systems. (2) A consensus should be reached within the scientific community regarding definitions for the design of test data and quality criteria for the evaluation and comparability of DDx systems. Furthermore, open source thesauri for histopathological morphology, radiological findings and laboratory data should be included in medical decision support systems. Furthermore, the use of real world queries of physicians in two different online plattforms indicates that focusing on case reports to evaluate DDx systems is probably not the last word in truth. Here, the systems perform best on average, but case reports reflect the clinical situation at the primary patient presentation only to a very limited extent, since they were created ex post. Therefore, an actual clinical, prospective study using DDx and retrospective evaluation of primary outcomes would be highly desirable to test the actual use case.

## Conclusion

In summary, both DDx systems IsabelHealth and Memem7 provided substantial help in finding a helpful list of differential diagnoses or identifying the target diagnosis either in standard cases or complicated and rare cases. Nevertheless, finding suitable test procedures or standards to test and validate holistic DDx remains a major and complex challenge. Our pilot study highlights the need for different levels of complexity and types of real-world medical test cases, as there are significant differences between DDx generators away from traditionally employed case reports. The comparison of concrete different results of the DDx generators and, if necessary, the combination of different DDx systems seems to be a possible approach for future review and use. DDx do hold a great promise for further use in medicine.

## Supplementary Information

**Additional file 1**: **Table S1**. List of the cases taken from the New England Journal of Medicine. **Table S2**. Legend for the file "Dataset.csv". This file contains the extracted keywords (here referred to as ‘causophemes’) both in German, if the original cases were in German, and their used translation and the target diagnoses.

## Data Availability

The datasets supporting the conclusions of this article are included within the article and its additional files; study protocol, statistical analysis plan, etc. will be shared upon request to the correspondence author (namely Severin Schricker) and after review of institutional privacy policies. Published cases can be accessed at the references published in the additional file (NEJM, Additional file 1: Table S1) or at https://www.ipath-network.com (Afghan cases) and https://www.coliquio.de/ (Coliquio cases). The used dataset of extracted keywords and target diagnoses of all test cases can also be found in the additional file (a legend can be found in the Additional file 1: Table S2).
