# Supplementary material for: Evaluation of medical decision support systems (DDX generators) using real medical cases of varying complexity and origin
Source: BMC Med Inform Decis Mak. 2022 Sep 24;22:254. doi: 10.1186/s12911-022-01988-2 (PMC9509605; doi:10.1186/s12911-022-01988-2)
Supplement: Supplementary file 1 — Additional file 1: Table S1. List of the cases taken from the New England Journal of Medicine. Table S2. Legend for the file "Dataset.csv". This file contains the extracted keywords (here referred to as ‘causophemes’) both in German, if the original cases were in German, and their used translation and the target diagnoses. [file 12911_2022_1988_MOESM1_ESM.docx]

**Supplementary material**

**Supplementary table 1. List of the cases taken from the New England Journal of Medicine.**

| **ID** | **NEJM Case** |
| --- | --- |
| 1 | Eichler, F.S., et al., Case 38-2017. A 20-Year-Old Woman with Seizures and Progressive Dystonia. N Engl J Med, 2017. 377(24): p. 2376-2385. |
| 2 | Raja, A.S., et al., Case 37-2017. A 36-Year-Old Man with Unintentional Opioid Overdose. N Engl J Med, 2017. 377(22): p. 2181-2188. |
| 3 | Sykes, D.B., et al., Case 40-2017. A 32-Year-Old Woman with Headache, Abdominal Pain, Anemia, and Thrombocytopenia. N Engl J Med, 2017. 377(26): p. 2581-2590. |
| 4 | Tsiaras, S.V., et al., Case 39-2017. A 41-Year-Old Woman with Recurrent Chest Pain. N Engl J Med, 2017. 377(25): p. 2475-2484. |
| 5 | Ard, K.L., et al., Case 9-2018: A 55-Year-Old Man with HIV Infection and a Mass on the Right Side of the Face. N Engl J Med, 2018. 378(12): p. 1143-1152. |
| 6 | Cestari, D.M., et al., Case 2-2018. A 41-Year-Old Woman with Vision Disturbances and Headache. N Engl J Med, 2018. 378(3): p. 282-289. |
| 7 | Chan, Y.M., R. Balza, and F.A. High, Case 3-2018: A 5-Month-Old Boy with Hypoglycemia. N Engl J Med, 2018. 378(4): p. 381-389. |
| 8 | Coutifaris, C., et al., Case 29-2018: A 31-Year-Old Woman with Infertility. N Engl J Med, 2018. 379(12): p. 1162-1172. |
| 9 | Dahl, D.M., et al., Case 36-2018: A 29-Year-Old Man with an Incidentally Discovered Renal Mass. N Engl J Med, 2018. 379(21): p. 2064-2072. |
| 10 | El-Jawahri, A.R., et al., Case 5-2018: A 63-Year-Old Man with Confusion after Stem-Cell Transplantation. N Engl J Med, 2018. 378(7): p. 659-669. |
| 11 | Falk, R.J., et al., Case 24-2018: A 71-Year-Old Man with Acute Renal Failure and Hematuria. N Engl J Med, 2018. 379(6): p. 568-578. |
| 12 | Farber, H.W., et al., Case 11-2018: A 48-Year-Old Woman with Recurrent Venous Thromboembolism and Pulmonary Artery Aneurysm. N Engl J Med, 2018. 378(15): p. 1430-1438. |
| 13 | Goldstein, J.N., et al., Case 12-2018: A 30-Year-Old Woman with Cardiac Arrest. N Engl J Med, 2018. 378(16): p. 1538-1549. |
| 14 | Hibbert, K.A., et al., Case 1-2018. A 39-Year-Old Woman with Rapidly Progressive Respiratory Failure. N Engl J Med, 2018. 378(2): p. 182-190. |
| 15 | Isselbacher, E.M., et al., Case 38-2018: A 54-Year-Old Man with New Heart Failure. N Engl J Med, 2018. 379(24): p. 2362-2372. |
| 16 | Iyasere, C.A., et al., Case 28-2018: A 39-Year-Old Man with Epistaxis, Pain and Erythema of the Forearm, and Pancytopenia. N Engl J Med, 2018. 379(11): p. 1072-1081. |
| 17 | Knuesel, S.J., et al., Case 6-2018: A 35-Year-Old Woman with Headache, Subjective Fever, and Anemia. N Engl J Med, 2018. 378(8): p. 753-760. |
| 18 | Kobayashi, K.J., A.A. Weil, and J.A. Branda, Case 16-2018: A 45-Year-Old Man with Fever, Thrombocytopenia, and Elevated Aminotransferase Levels. N Engl J Med, 2018. 378(21): p. 2023-2029. |
| 19 | Lefebvre, D.R., et al., Case 39-2018: An 18-Year-Old Man with Diplopia and Proptosis of the Left Eye. N Engl J Med, 2018. 379(25): p. 2452-2461. |
| 20 | Loscalzo, J., et al., Case 8-2018: A 55-Year-Old Woman with Shock and Labile Blood Pressure. N Engl J Med, 2018. 378(11): p. 1043-1053. |
| 21 | Mojica, J.E., et al., Case 40-2018: A 47-Year-Old Woman with Recurrent Sinusitis, Cough, and Bronchiectasis. N Engl J Med, 2018. 379(26): p. 2558-2565. |
| 22 | Morris, C.A., et al., Case 17-2018: A 40-Year-Old Woman with Leg Swelling and Abdominal Distention and Pain. N Engl J Med, 2018. 378(22): p. 2124-2132. |
| 23 | Neilan, T.G., et al., Case 33-2018: A 57-Year-Old Man with Confusion, Fever, Malaise, and Weight Loss. N Engl J Med, 2018. 379(17): p. 1658-1669. |
| 24 | Paras, M.L., et al., Case 14-2018: A 68-Year-Old Woman with a Rash, Hyponatremia, and Uveitis. N Engl J Med, 2018. 378(19): p. 1825-1833. |
| 25 | Prasad, S., et al., Case 37-2018: A 23-Year-Old Woman with Vision Loss. N Engl J Med, 2018. 379(22): p. 2152-2159. |
| 26 | Reda, H.M., et al., Case 34-2018: A 58-Year-Old Woman with Paresthesia and Weakness of the Left Foot and Abdominal Wall. N Engl J Med, 2018. 379(19): p. 1862-1868. |
| 27 | Rubin, A.K., et al., Case 30-2018: A 66-Year-Old Woman with Chronic Abdominal Pain. N Engl J Med, 2018. 379(13): p. 1263-1272. |
| 28 | Sacks, C.A., et al., Case 20-2018: A 64-Year-Old Man with Fever, Arthralgias, and Testicular Pain. N Engl J Med, 2018. 378(26): p. 2518-2529. |
| 29 | Taylor, J.B., et al., Case 21-2018: A 61-Year-Old Man with Grandiosity, Impulsivity, and Decreased Sleep. N Engl J Med, 2018. 379(2): p. 182-189. |
| 30 | Thompson, R.W., et al., Case 10-2018: An 84-Year-Old Man with Painless Unilateral Testicular Swelling. N Engl J Med, 2018. 378(13): p. 1233-1240. |
| 31 | Turbett, S.E., et al., Case 26-2018: A 48-Year-Old Man with Fever, Chills, Myalgias, and Rash. N Engl J Med, 2018. 379(8): p. 775-785. |
| 32 | Wexler, D.J., et al., Case 23-2018: A 36-Year-Old Man with Episodes of Confusion and Hypoglycemia. N Engl J Med, 2018. 379(4): p. 376-385. |
| 33 | Ziperstein, J.C., et al., Case 13-2018: A 53-Year-Old Man with Cardiomyopathy and Recurrent Ventricular Tachycardia. N Engl J Med, 2018. 378(17): p. 1622-1633. |
| 34 | Arvikar, S.L., et al., Case 17-2019: A 44-Year-Old Man with Joint Pain, Weight Loss, and Chest Pain. N Engl J Med, 2019. 380(22): p. 2157-2167. |
| 35 | Black, K.E., et al., Case 10-2019: A 69-Year-Old Man with Progressive Dyspnea. N Engl J Med, 2019. 380(13): p. 1268-1277. |
| 36 | Chen, J.A., et al., Case 9-2019: A 62-Year-Old Man with Atrial Fibrillation, Depression, and Worsening Anxiety. N Engl J Med, 2019. 380(12): p. 1167-1174. |
| 37 | Chen, S.T., et al., Case 2-2019: A 36-Year-Old Man with Rash, Abdominal Pain, and Lymphadenopathy. N Engl J Med, 2019. 380(3): p. 275-283. |
| 38 | Donovan, A.L., et al., Case 1-2019: A 34-Year-Old Veteran with Multiple Somatic Symptoms. N Engl J Med, 2019. 380(2): p. 178-185. |
| 39 | Doughty, C.T., et al., Case 12-2019: A 60-Year-Old Man with Weakness and Difficulty Chewing. N Engl J Med, 2019. 380(16): p. 1566-1574. |
| 40 | Hogan, C., et al., Case 5-2019: A 48-Year-Old Woman with Delusional Thinking and Paresthesia of the Right Hand. N Engl J Med, 2019. 380(7): p. 665-674. |
| 41 | Ison, M.G., et al., Case 20-2019: A 52-Year-Old Woman with Fever and Rash after Heart Transplantation. N Engl J Med, 2019. 380(26): p. 2564-2573. |
| 42 | Khalili, H., et al., Case 19-2019: A 38-Year-Old Woman with Abdominal Pain and Fever. N Engl J Med, 2019. 380(25): p. 2461-2470. |
| 43 | Rosenbaum, J.T., et al., Case 8-2019: A 58-Year-Old Woman with Vision Loss, Headaches, and Oral Ulcers. N Engl J Med, 2019. 380(11): p. 1062-1071. |
| 44 | Schaefer, E.A., et al., Case 15-2019: A 55-Year-Old Man with Jaundice. N Engl J Med, 2019. 380(20): p. 1955-1963. |
| 45 | Shields, H.M., et al., Case 4-2019: An 18-Year-Old Man with Abdominal Pain and Hematochezia. N Engl J Med, 2019. 380(5): p. 473-485. |
| 46 | Simmons, R.P., et al., Case 16-2019: A 53-Year-Old Man with Cough and Eosinophilia. N Engl J Med, 2019. 380(21): p. 2052-2059. |
| 47 | Soumerai, J.D., et al., Case 7-2019: A 73-Year-Old Woman with Swelling of the Right Groin and Fever. N Engl J Med, 2019. 380(9): p. 859-868. |
| 48 | Spriggs, D.R., et al., Case 18-2019: A 24-Year-Old Woman with a Pelvic Mass. N Engl J Med, 2019. 380(24): p. 2361-2369. |
| 49 | Walley, A.Y., S.E. Wakeman, and G. Eng, Case 6-2019: A 29-Year-Old Woman with Nausea, Vomiting, and Diarrhea. N Engl J Med, 2019. 380(8): p. 772-779. |
| 50 | Zachary, K.C., et al., Case 3-2019: A 70-Year-Old Woman with Fever, Headache, and Progressive Encephalopathy. N Engl J Med, 2019. 380(4): p. 380-387. |

**Supplementary table 2. Legend for the file "Dataset.csv".** This file contains the extracted keywords (here referred to as ‘causophemes’) both in German, if the original cases were in German, and their used translation and the target diagnoses.

| **List of the occurring items** | **Description** |
| --- | --- |
| Identifier | identifier that uniquely identifies the cases |
| Origin | identifies the case as either NEJM, Coliquio or Afghan case |
| age |  |
| sex |  |
| kasuophem 1 – 12 | extracted keywords in German where appropriate |
| English_Kasu_1 - 12 | translated extracted keywords where appropriate |
| Target diagnosis | extracted target diagnoses in German where appropriate |
| Target diagnosis English | translated extracted target diagnosis where appropriate |
